# Treatment of an acutely ruptured complex fusiform middle cerebral artery aneurysm with flow diverting stenting and adjunctive coil embolization

**DOI:** 10.3171/2022.7.FOCVID2249

**Published:** 2022-10-01

**Authors:** Guilherme Barros, Michael R. Levitt

**Affiliations:** Departments of 1 Neurological Surgery,; 2 Radiology,; 3 Mechanical Engineering, and; 4 Stroke & Applied Neuroscience Center, University of Washington, Seattle, Washington

**Keywords:** flow-diverting stent, flow diversion, middle cerebral artery aneurysm, coil embolization, subarachnoid hemorrhage

## Abstract

This technical video demonstrates the treatment of an acutely ruptured, large, complex left fusiform middle cerebral artery (MCA) aneurysm with endovascular flow diversion with adjunctive coiling in a 27-year-old female. Two telescoping flow-diverting stents (Pipeline Flex) were placed, with partial coiling of a saccular portion of the aneurysm. Technical challenges, alternative treatment, intraoperative and postoperative antiplatelet management, vasospasm treatment, and clinical and radiographic follow-up are described.

The video can be found here: https://stream.cadmore.media/r10.3171/2022.7.FOCVID2249

## Transcript

Treatment of an acutely ruptured complex fusiform middle cerebral artery aneurysm with flow-diverting stenting and adjunctive coil embolization.

### 0:33 Case Description.

A 27-year-old female presented with sudden-onset severe headache and loss of consciousness. Noncontrast head CT demonstrated extensive subarachnoid hemorrhage, and CTA identified a ruptured fusiform left MCA aneurysm. A ventriculostomy was placed and she was referred for catheter angiography and embolization.^1–10^

### 0:53 Initial Diagnostic, Left Internal Carotid Artery (ICA), AP/Lateral.

The right wrist was prepped and draped in a sterile fashion and a 22-gauge needle was used to puncture the right radial artery with ultrasound guidance. A 7-Fr Merit Prelude Ideal slender sheath was advanced over wire and secured in place. A radial cocktail of 2.5 mg of verapamil and 200 µg of nitroglycerin was administered intra-arterially, and the patient was fully heparinized with an activated clotting time (ACT) of greater than 250 seconds. A 5-Fr Simmons II glide catheter was advanced over the wire and navigated into the left ICA, and angiographic views demonstrating the large left fusiform MCA aneurysm were obtained.

### 1:38 Initial Diagnostic, Left ICA 3D Reconstruction With Stent Planning.

Three-dimensional rotational angiography demonstrated a multilobulated and complex aneurysm. The estimated rupture point was the saccular component measuring 17 mm right to left, 12.36 mm craniocaudal, and 13.2 mm anterior-posterior. There was significant dysplasia along the entire distal M1 segment and proximal M2 segments of the MCA, with several lateral lenticulostriate branches arising from the proximal aspect of the aneurysm wall. A stent plan was then measured on the 3D reconstruction.

### 2:16 Catheterization of Left MCA, AP/Lateral.

The 7-Fr slender sheath was exchanged over a wire for an 088 Infinity long sheath. Then, a 4-Fr 125-cm vert catheter was placed through the Infinity and brought into the left ICA over a Glidewire. The Infinity sheath was then advanced up to the distal straight segment of the cervical left ICA, and the 4-Fr catheter was withdrawn. An 072 Navien intermediate catheter was placed over a Phenom Plus intermediate catheter for additional support, which was placed over a Phenom 27 microcatheter and Synchro II microwire. Using this system, the microcatheter was navigated across the aneurysm dome and into a distal M3 branch. The Navien catheter was advanced to the left MCA origin. The Phenom Plus and Phenom 27 were removed, and the Phenom 27 was again navigated across the aneurysm dome into the left M3 branch. An Echelon 10 microcatheter was placed into the aneurysm dome for jailing and subsequent partial coiling.

### 3:19 Poststent Placement, Left ICA, AP/Lateral/Stent-View CT.

The first stent, a 4.25 × 35–mm Pipeline Flex, was deployed from the superior division M2 to the distal M1 segment, partially spanning the aneurysm dome and jailing the inferior division M2. Because the proximal aspect of the stent did not adequately cover the entirety of the aneurysm, the Phenom 27 was navigated through the stent, and a second Pipeline Flex, 4.5 × 25 mm, was telescoped from the largest portion of the aneurysm dome into the distal carotid terminus. The microcatheter was navigated back through this stent construct, and contrast-enhanced flat-panel CT was obtained, which demonstrated good proximal and distal wall apposition and complete aneurysm coverage. A weight-based half-loading dose of 12 mg of eptifibatide was injected intra-arterially into the left MCA via the Navien catheter for immediate antiplatelet effect after stent placement.

### 4:15 Postcoil, Left MCA, AP/Lateral.

Two Target XL 360 soft coils were placed through the jailed Echelon microcatheter into the putative rupture point of the aneurysm. While it is thought that coils provide additional substrate for aneurysm thrombosis, especially in large, ruptured aneurysms treated with flow diversion, we purposefully loosely coiled the aneurysm to prevent significant thrombosis of any perforators. The microcatheters were removed, and subsequent angiographic views demonstrated good embolization of the aneurysm with flow stasis in the aneurysm dome and preserved flow through daughter branches.

### 4:49 Final Postintervention Runs, Left ICA, AP/Lateral.

Final left ICA AP/lateral angiography shows that after overlapping flow-diverting stent placement, there is contrast stasis in the aneurysm dome, with reduced flows yet further in the coiled portion of the aneurysm dome and preservation of normal vasculature. Immediately postoperatively, the patient was loaded on 650 mg of aspirin and 180 mg of ticagrelor, followed by aspirin 81 mg daily and ticagrelor 90 mg twice daily.

### 5:21 Four Days Postintervention, Left ICA, AP/Lateral.

Four days following the intervention, an angiogram was obtained to evaluate for potential cerebral vasospasm. The AP/lateral diagnostic views demonstrated interval remodeling of the M2 branches and partial thrombosis of the aneurysm with occlusion of the presumed rupture point.

### 5:42 Four Weeks Postintervention, Left ICA, AP/Lateral.

Four weeks following the intervention, another angiogram was obtained to evaluate for potential cerebral vasospasm. There was continued remodeling of the aneurysmal segment of the left MCA with no significant branch vessel occlusion, and significant reduction in aneurysm dome size, similar to the prior angiogram.

### 6:04 6 Months Postintervention, Left ICA, AP/Lateral/Oblique/3D Reconstruction.

Six months following the intervention, an outpatient follow-up angiogram was performed. This demonstrated continued remodeling of the aneurysm, with stent patency. There is significant remodeling of the inferior aspect of the MCA as well. There is still fullness to the patient’s MCA bifurcation, but no obvious new aneurysmal development, and there is branch patency of all distal MCA branches.

### 6:40 Antiplatelet Management.

The patient was loaded with 650 mg of aspirin and 180 mg of ticagrelor immediately following the intervention. She was then maintained on 81 mg of aspirin once daily and 90 mg of ticagrelor twice daily for 6 months postintervention. The patient failed her external ventricular drain wean and required placement of a ventriculoperitoneal shunt for long-term hydrocephalus management. We maintained her dual antiplatelet therapy regimen for the operation and used her existing ventriculostomy catheter as the proximal shunt catheter to avoid intracranial manipulation. Laparoscopic distal shunt catheter placement was performed by general surgery. If the patient were to have instead successfully weaned the ventriculostomy without the need for shunt placement, we would have ligated the ventricular catheter in situ in the operating room with sutures to prevent any further drainage of cerebrospinal fluid. We then would secure it to the skull with a cranial plate and screws to prevent displacement of the catheter. This avoids the risk of catheter tract hemorrhage during removal while on dual antiplatelet therapy. At 6 months following her intervention, the ticagrelor was discontinued and she continued aspirin 81 mg once daily long-term.

### 7:54 Alternative Treatment.

Alternative treatment of the aneurysm would be microsurgical trapping and a double-barrel bypass given the complex morphology and involvement of distal MCA branches. There is a high stroke risk and surgical morbidity associated with such an operation, due to direct occlusion of MCA perforators, and thus an endovascular approach was chosen to minimize these risks. Flow-diverting stent treatment should maintain significant parent vessel perforator and side branch patency in most treatments. However, we recognize there is still a risk for perforators arising from the aneurysm dome to occlude when the aneurysm thromboses. To promote thrombosis of the large, saccular portion of this aneurysm and minimize the risk of perforator occlusion, we purposefully loosely coiled the aneurysm.

## Acknowledgments

We thank James Pan, MD, for his assistance with formatting of angiographic images for video preparation.

## Disclosures

Dr. Levitt: recipient of unrestricted educational grants from Stryker and Medtronic; equity interest in Synchron, Cerebrotech, Proprio, Hyperion Surgical, and Fluid Biomed; advisor for Metis Innovative; and consultant for Medtronic and Aeaean Advisers.

## Author Contributions

Primary surgeon: Levitt. Assistant surgeon: Barros. Editing and drafting the video and abstract: both authors. Critically revising the work: both authors. Reviewed submitted version of the work: both authors. Approved the final version of the work on behalf of both authors: Levitt. Supervision: Levitt.
